# Sleep Deprivation Disturbs Immune Surveillance and Promotes the Progression of Hepatocellular Carcinoma

**DOI:** 10.3389/fimmu.2021.727959

**Published:** 2021-09-03

**Authors:** Jing Huang, Peiwen Song, Kaibin Hang, Zeka Chen, Zidan Zhu, Yuye Zhang, Jietian Xu, Jie Qin, Binghua Wang, Weimin Qu, Zhili Huang, Chunmin Liang

**Affiliations:** ^1^Laboratory of Tumor Immunology, Department of Anatomy, Histology, and Embryology, School of Basic Medical Sciences, Shanghai Medical College, Fudan University, Shanghai, China; ^2^Department of Neurobiology, School of Basic Medical Sciences, Fudan University, Shanghai, China; ^3^Department of Radiology, Naval Medical Center of People’s Liberation Army, Shanghai, China; ^4^Department of Pharmacology, School of Basic Medical Sciences, State Key Laboratory of Medical Neurobiology and Ministry of Education (MOE) Frontiers Center for Brain Science, and Institutes of Brain Science, Fudan University, Shanghai, China

**Keywords:** sleep deprivation, hepatocellular carcinoma, tumor microenvironment, CD3^+^ T cells, NK cell, CD11b^+^ cell

## Abstract

Sleep disturbance is common in patients with cancer and is associated with poor prognosis. However, the effects of sleep deprivation (SD) on immune surveillance during the development of hepatocellular carcinoma (HC) and the underlying mechanisms are not known. This was investigated in the present study using mouse models of SD and tumorigenesis. We determined that acute and chronic sleep deprivation (CSD) altered the relative proportions of various immune cell types in blood and peripheral organs. CSD increased tumor volume and weight, an effect that was enhanced with increasing CSD time. Expression of the cell proliferation marker Ki-67 was elevated in tumor tissues, and tumor cell infiltration into adjacent muscles was enhanced by CSD. Multicolor flow cytometry analysis revealed that CSD significantly reduced the numbers of antitumor CD3^+^ T cells and natural killer (NK) cells and increased that of immunosuppressive CD11b^+^ cells infiltrating into the tumor microenvironment from the spleen *via* the peripheral blood. These results indicate that CSD impairs immune surveillance mechanisms and promotes immunosuppression in the tumor microenvironment to accelerate tumor growth, underscoring the importance of alleviating sleep disturbance in HC patients in order to prevent HC progression.

## Introduction

Sleep deprivation (SD), which is defined as getting insufficient sleep, has adverse health effects and has been linked to various pathologies including cardiovascular disease, cancer, diabetes, kidney disease, stroke, and mood disorders (1, 2), and a compromised immune response (3). SD induces changes in immune indices and functions including lymphocyte migration and distribution, cytokine production, immunoglobulin G level, antigen presentation, and complement activation (4), which are associated with inflammatory diseases (5). A single night of acute sleep deprivation (ASD) significantly altered the number and function of circulating immature neutrophils in healthy men, which can partly explain why people experiencing poor sleep are more prone to infections (6). However, most research on the pathological effects of SD have analyzed human peripheral blood samples, which does not provide a comprehensive view of physiological changes. Meanwhile, there have been no detailed studies on SD-induced changes in the immune system, and the few findings on this topic are controversial because of the different models of SD that were used.

Chronic inflammation involving immune cell subsets promotes tumor initiation, progression, and metastasis (7), which is referred to as cancer-associated inflammation (8). The tumor microenvironment harbors various immune cell types such as CD3^+^ T lymphocytes, CD4^+^ T helper cells (Th), cytotoxic T cells (CD8^+^), natural killer (NK) cells, and natural killer T (NKT) cells along with CD11b^+^ myeloid cell subsets including myeloid-derived suppressor cells (MDSCs) and tumor-associated macrophages (9). An increased number of tumor-infiltrating CD3^+^ T lymphocytes was shown to be correlated with good prognosis in advanced ovarian cancer (10). Among CD4^+^ T cells subsets, Th1 cells are the major antitumor effectors, whereas Th2 cells and regulatory T lymphocytes (Tregs) are immunosuppressive and inhibit the antitumor immune response. CD8^+^ T cells and NK cells also have antitumor immune effector functions (11, 12). Myeloid cells are immunosuppressive and promote tumor development. As immature myeloid cells, MDSCs in mice are defined as CD11b^+^GR1^+^ and inhibit the generation and function of NK cells and T cells. Accumulated MDSCs promote tumor growth and metastasis, which reduces the efficacy of anticancer therapies in patients (13).

Epidemiological studies indicate that sleep disturbance is common in patients with cancer (14), which is linked to worse prognosis and decreased quality of life (15). As the fourth leading cause of cancer death worldwide (16), hepatocellular carcinoma (HC) usually begins as an unresolved inflammatory response to infection with hepatitis B virus (HBV) that develops into liver cirrhosis and cancer (17). There is evidence that sleep disorders are linked to increased liver cancer risk (18) and mortality (19); however, to date there have been no studies investigating the effects of CSD on HC progression and the associated immunological factors.

We speculated that CSD-induced changes in the immune system contribute to HC development and progression. To test this hypothesis, we used well-established mouse models of ASD and CSD to mimic the long-term sleep disturbance experienced by cancer patients. Moderate SD was induced by depriving mice of sleep for 6 h/day, which did not cause stress or hyperalgesia, thus eliminating the influence of these factors (20). We examined the impact of ASD and CSD on the immune profiles of mice. We also explored the effects of CSD on HC progression using a subcutaneous tumor-bearing mouse model and examined whether SD resulted in dysregulation and redistribution of immune cells in the tumor microenvironment and peripheral immune organs (spleen and lymph node).

## Materials and Methods

### Cell Lines and Mice

Hep1-6 mouse hepatoma cells were used to establish a mouse HC model in previous study of our lab (21). In addition, the histological features of HC models built by Hep1-6 cells in experimental animals are highly similar to those in clinical patients (22). Thus, Hep1-6 cells were used in this study, which were obtained from the Cell Bank of the Chinese Academy of Sciences (Shanghai, China; #TCM39) and cultured in Dulbecco’s modified Eagle’s medium (Gibco, Grand Island, NY, USA) containing 10% fetal bovine serum (Gibco) in a 37°C incubator with 5% CO_2_. Five-week-old male C57BL/6J mice were purchased from Shanghai SLAC Laboratory Animal Co. (Shanghai, China) and housed six per cage. The mice had free access to food and water and were maintained on a 12:12-h light/dark cycle (lights on at 07:00 a.m.) in a temperature-controlled environment.

### ASD and CSD

The protocols for SD were based on a previous study (20). All mice were placed in the SD instrument for 30 min/day for 7 days so that they could adapt to the new environment prior to the experiment, thus eliminating potential interference from stress. SD was induced by placing a mouse on an automated moving platform. Once it started to sleep, the platform belt was moved so that the mouse ran into the surrounding barrier and were thus awakened. The normal sleep group received the same treatment but without the belt moving so that they could continue to sleep. During this period, mice could move freely and had access to food and water.

For the ASD model, mice were randomly divided into two groups: ASD (SD) and normal sleep [control (Con)]. ASD was induced by placing mice on the platform for 9 h (from 07:00 to 16:00). For the CSD model, mice were randomly divided into the CSD and normal sleep (Con) groups. The CSD group was subjected to partial SD for 6 h/day (from 7:00 a.m. to 13:00 p.m.) for 14 days. Tumor-bearing mice were divided into three groups: mice without CSD (T), mice subjected to CSD after tumor implantation (T + CSD), and mice subjected to CSD before and after tumor implantation (CSD + T + CSD).

### Tumor Models

Hep1-6 tumor cells [10^6^ in 200 µl serum-free Roswell Park Memorial Institute (RPMI)-1640 medium per mouse] were subcutaneously injected into the left flank of mice, which were then returned to their home cage. At 14 days after the injection, the mice were anesthetized with isoflurane, and a peripheral blood sample was collected by cardiac puncture. The mice were euthanized and subcutaneous tumors were enucleated and weighed. The spleen and mesenteric lymph nodes were dissected and preserved until use, when they were dissociated into a single-cell suspension. Tumor length (L) and width (W) were measured with Vernier calipers and used to calculate the tumor volume (V) with the following formula: V = (π/6) × L × W^2^. The tumor tissue was preserved in RPMI-1640 medium for flow cytometry analysis and fixed in 4% paraformaldehyde for sectioning.

### Hematoxylin and Eosin Staining

Tumor tissue samples from mice were fixed in 4% paraformaldehyde, dehydrated in a graded series of alcohol, and embedded in paraffin, then cut into 5-μm-thick sections that were deparaffinized, rehydrated, washed with double-distilled water, and stained with hematoxylin for 8 min. They were then rewashed in water, differentiated in 1% acid alcohol, and rinsed under flowing water. After staining with eosin for 3 min, the sections were dehydrated, cleared in dimethylbenzene, and mounted with neutral balsam. The stained samples were observed under a light microscope (Olympus, Tokyo, Japan) to assess tumor cell infiltration into muscle tissue.

### Immunofluorescence Analysis

Paraffin-embedded sections of tumor tissue were deparaffinized in dimethylbenzene and rehydrated in gradient alcohol. Antigen retrieval was performed, and sections were blocked with 5% goat serum at room temperature for 1 h, then labeled overnight at 4°C with primary antibody. The following day, the sections were washed three times with phosphate-buffered saline (PBS) and then incubated in Alexa Fluor 594-conjugated secondary antibody (1:500; Abcam, Cambridge, MA, USA). Cell nuclei were stained with 4′,6-diamidino-2-phenylindole at room temperature for 30 min. Images were acquired with a confocal laser scanning microscope. The number of Ki-67-positive cells in tumor tissue was counted using ImageJ software (National Institutes of Health, Bethesda, MD, USA).

### Flow Cytometry Analysis

Peripheral blood samples from mice were collected in anticoagulant tubes. Erythrocytes were lysed with lysis buffer (BD Biosciences, Franklin Lakes, NJ, USA), and the remaining cells were washed with 2 ml PBS. Spleen and lymph nodes were mechanically disrupted and passed through a 70-μm membrane filter to obtain single-cell suspensions that were washed with RPMI-1640 medium and labeled with the following antibodies: CD45-V500, CD3-allophycocyanin (APC)-Cy7, CD4-fluorescein isothiocyanate, CD8-peridinin chlorophyll protein-Cy5.5, NK1.1-BV650, CD11b-BV421, GR1-BV605, Ly6G-phycoerythrin (PE)-Cy7, Ly6C-APC, and F4/80-PE (BD Biosciences). Cells were sorted by flow cytometry (LSRFortessa; BD Biosciences), and the data were analyzed with FlowJo software (Tree Star, Ashland, OR, USA).

### Statistical Analysis

Results are presented as mean ± SEM. The threshold for statistical significance was a two-tailed p < 0.05. Differences in the proportions of immune cells between the ASD and CSD groups and their respective control groups were evaluated with the independent-samples t-test or non-parametric test depending on whether the data were normally distributed. Tumor volume and weight and immune cells in blood, spleen, lymph node, and tumors in tumor-bearing mice were assessed by one-way analysis of variance followed by the multiple comparisons test. All statistical analyses were performed using Prism v7.0 software (GraphPad, La Jolla, CA, USA).

## Results

### ASD Alters the Ratios of Immune Cell Types

To investigate the impact of sleep disturbance on immune function, we established mouse ASD and CSD models and used multicolor flow cytometry to detect the proportions of immune cells in blood and peripheral immune organs (spleen and lymph node) in these mice compared to control mice (Con).

**Figure 1:**
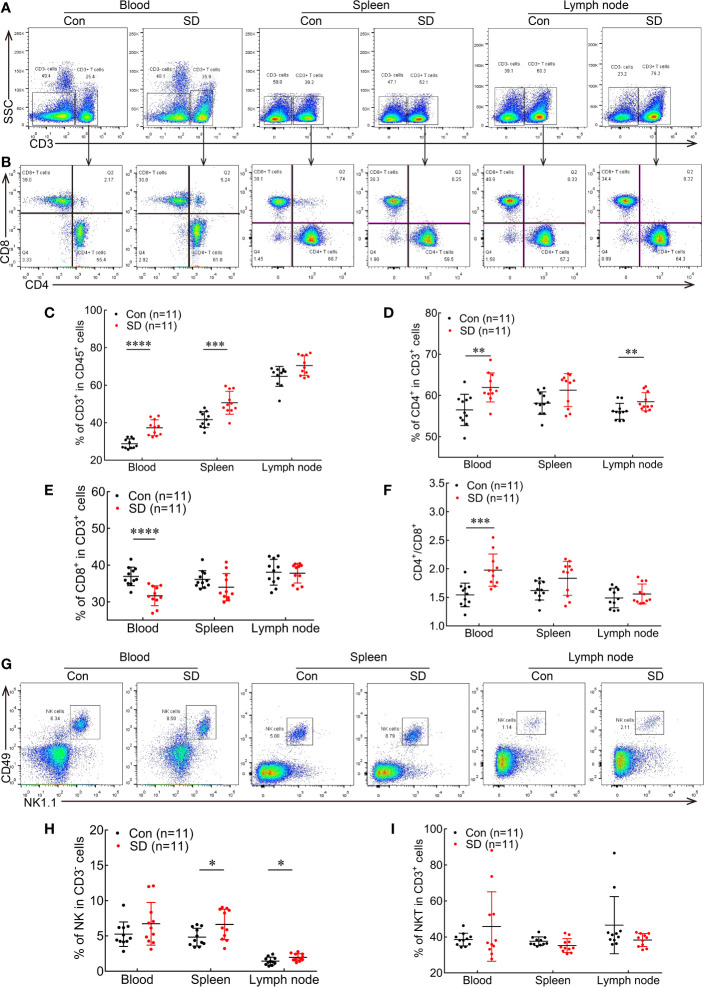
ASD alters the proportions of immune cells in the peripheral blood, spleen, and lymph node. **(A, B)** Flow cytometry plots showing CD3^+^ T cells **(A)**, CD4^+^ T cells, and CD8^+^ T cells **(B)** in blood, spleen, and lymph node in ASD (SD) and normal sleep (Con) groups. **(C–F)** Statistical analysis of the percentages of CD3^+^ T cells **(C)**, CD4^+^ T cells **(D)**, and CD8^+^ T cells **(E)**, and CD4/CD8 ratio **(F)** in the circulation and peripheral immune organs of SD and Con groups. **(G)** Representative dot plots of NK cells in peripheral blood, spleen, and lymph node in the SD and Con groups. **(H, I)** Statistical analysis of the proportions of NK cells **(H)** and NKT cells **(I)** in SD and Con groups (n = 11 per group). Data are presented as mean ± SEM. *p < 0.05, **p < 0.01, ***p < 0.001, ****p < 0.0001.

We first examined the impact of ASD on T lymphocytes in peripheral blood, spleen, and lymph node (**Figures 1A–F**). After 9 h of SD, the percentage of CD3^+^ T lymphocytes among CD45^+^ cells was significantly increased in peripheral blood compared to the Con group (37.3 ± 1.275 *vs*. 28.75 ± 0.776, *p* < 0.0001; **Figure 1C**). The number of CD4^+^ T cells was significantly increased in the blood among CD3^+^ T cell subsets (61.95 ± 1.064 *vs*. 56.49 ± 1.139, *p* = 0.0023; **Figure 1D**) along with the CD4/CD8 ratio (1.977 ± 0.0854 *vs*. 1.542 ± 0.0622, *p* = 0.0005; **Figure 1F**), while the percentage of cytotoxic CD8^+^ T cells among CD3^+^ T cells in the peripheral blood was lower compared to mice without SD (Con) (31.67 ± 0.8126 *vs*. 36.93 ± 0.7624, *p* = 0.0001; **Figure 1E**). In the spleen, ASD markedly increased the proportion of CD3^+^ T cells compared to the Con group (50.63 ± 1.831 *vs*. 41.65 ± 1.27, *p* = 0.0007; **Figure 1C**). The percentage of CD4^+^ T cells (**Figure 1D**) and CD4/CD8 ratio (**Figure 1F**) were higher, whereas the percentage of CD8^+^ T cells showed a decreasing trend after ASD (**Figure 1E**), although the difference was non-significant. In the lymph node, the percentage of CD4^+^ T cells was increased in the SD group (58.48 ± 0.6618 *vs*. 56.15 ± 0.5846, *p* = 0.007; **Figure 1D**). ASD also tended to increase the percentage of CD3^+^ T cells (**Figure 1C**) and CD4/CD8 ratio (**Figure 1F**) but did not affect the proportion of CD8^+^ T cells (**Figure 1E**) in the lymph node.

We also compared the abundance of NK and NKT cells in the blood, spleen, and lymph node between SD and Con groups (**Figures 1G–I**). No significant difference was observed in the proportions of NK cells among CD3^−^ cells (**Figure 1H**) and of NKT cells among CD3^+^ cells in the blood between the two groups. However, ASD increased the percentage of NK cells in the spleen (6.625 ± 0.653 *vs*. 4.828 ± 0.378, *p* = 0.0274; **Figure 1H**). Meanwhile, the percentage of NKT cells in the spleen showed a decreasing trend but did not differ significantly between the SD and Con groups (**Figure 1I**). The percentage of NK cells was also higher in the lymph node (1.967 ± 0.1556 *vs*. 1.435 ± 0.1652, *p* = 0.0294; **Figure 1H**), while NKT cells numbers did not differ between the SD and Con groups (**Figure 1I**). These results indicate that ASD causes similar alterations in different immune cell populations in blood and peripheral immune organs, thereby altering the systemic immune profile in mice.

### CSD Alters Immune Profiles in Blood, Spleen, and Lymph Node

**Figure 2:**
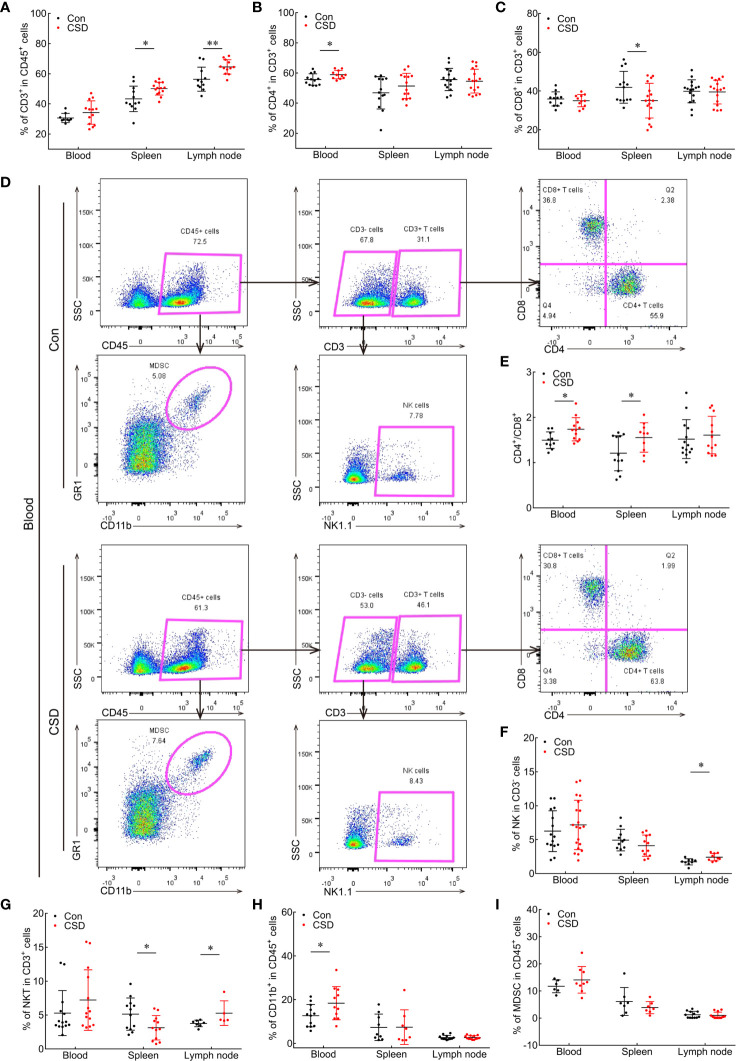
Effect of CSD on the proportions of immune cells in the peripheral blood, spleen, and lymph node of mice. **(A–C)** Quantification of CD3^+^ T cells **(A)**, CD4^+^ T cells **(B)**, and CD4/CD8 ratio **(C)** in the blood and peripheral immune organs. **(D)** Representative gating strategy in multicolor flow cytometry and scatterplots of immune cell subsets in the blood of CSD and Con groups. CD45^+^ cells were first gated for the total population of white blood cells. CD45^+^CD11b^+^GR1^+^ cells are presented as MDSCs; CD3^+^CD4^+^CD8^−^ and CD3^+^CD4^−^CD8^+^ cells were defined as CD4^+^ T cells and CD8^+^ T cells, respectively; and CD3^-^NK1.1^+^ cells were gated as NK cells. **(E–I)** Comparisons of CD4/CD8 ratio **(E)** and proportions of NK cells **(F)**, NKT cells **(G)**, CD11b^+^ cells **(H)**, and MDSCs **(I)** in the blood, spleen, and lymph node between CSD and Con groups. Data are presented as mean ± SEM. *p < 0.05, **p < 0.01.

To investigate the impact of CSD on the immune cell profile, mice were subjected to CSD for 6 h/day for 14 days. T lymphocytes, NK cells, NKT cells, and CD11b^+^ cell subsets in the blood and peripheral immune organs were detected by multicolor flow cytometry (**Figure 2D**). Compared to the group without SD (Con), CD3^+^ T cells in the blood of CSD mice showed an increasing trend, although the difference was non-significant. CSD markedly increased the percentage of CD3^+^ T cells in the spleen (50.14 ± 1.151 *vs*. 43.34 ± 2.444, *p* < 0.05) and lymph node (64.58 ± 1.382 *vs*. 56.42 ± 2.416, *p* = 0.0069) (**Figure 2A**). CD4^+^ T cells constituted the main subset of CD3^+^ cells in peripheral blood (58.95 ± 0.8432 *vs*. 55.77 ± 1.086, *p* < 0.05; **Figure 2B**). The percentage of CD8^+^ T cells was reduced in the spleen (35.04 ± 2.182 *vs*. 41.93 ± 2.285, *p* < 0.05; **Figure 2C**), while CD4/CD8 ratio in the blood (1.738 ± 0.074 *vs*. 1.495 ± 0.058, *p* < 0.05) and spleen (1.556 ± 0.103 *vs*. 1.206 ± 0.112, *p* < 0.05) were both increased (**Figure 2E**) in CSD mice.

We also analyzed changes in NK and NKT cell populations under CSD. Mice in this group had significantly higher numbers of NK cells (2.417 ± 0.182 *vs*. 1.733 ± 0.152, *p* < 0.05) and NKT cells (5.28 ± 0.809 *vs*. 3.763 ± 0.182, *p* < 0.05) in the lymph node and lower numbers of NKT cells in the spleen (3.123 ± 0.517 *vs*. 5.138 ± 0.684, *p* < 0.05) (**Figures 2F, G**) compared to the Con group, indicating that CSD alters T cell subsets, NK cells, and NKT cells to cause an imbalance in immune profiles in the peripheral blood, spleen, and lymph node of mice.

We also examined CD11b^+^ myeloid cell subsets in the blood and peripheral immune organs and found that CD11b^+^ was highly expressed on macrophages and MDSCs, which comprised two subpopulations: CD11b^+^Ly6G^+^Ly6C^low^ polymorphonuclear MDSCs (PMN-MDSCs) and CD11b^+^Ly6G^-^Ly6C^+^ monocytic MDSCs (M-MDSCs). We then assessed the influence of CSD on the percentages of myeloid cell subsets (MDSCs, PMN-MDSCs, M-MDSCs, and macrophages) and found a significantly higher proportion of CD11b^+^ myeloid cells among CD45^+^ cells in the blood of mice in the CSD group (18.42 ± 2.289 *vs*. 12.67 ± 1.472, *p* < 0.05), whereas the proportion was slightly decreased in the spleen (**Figure 2H**). MDSCs constituted the majority of CD11b^+^ cells that were significantly increased in peripheral blood and decreased in the spleen (**Figure 2I**), and PMN-MDSCs were the main subset of MDSCs that were increased in the blood and reduced in the spleen (**Supplementary Figure 1A**). In contrast, no changes were found in macrophage and M-MDSC populations in the blood and spleen of mice following CSD (**Supplementary Figures 1B, C**). Thus, CSD induces the mobilization of CD11b^+^ myeloid cells (mainly MDSCs and PMN-MDSCs) from spleen to circulation in C57BL/6 mice.

Taken together, our results indicate that ASD and CSD both altered the relative proportions of immune cells, which led to an imbalance in systemic immune profiles. Lymphocytes showed the same trend regardless of the tissue type (blood, spleen, and lymph node) or SD type (ASD and CSD). Specifically, ASD and CSD both increased the percentages of CD3^+^ T cells, CD4^+^ T cells, and NK cells and CD4/CD8 ratio and decreased the percentage of CD8^+^ T cells in the peripheral blood, spleen, and lymph node. Moreover, CSD may promote the mobilization of CD11b^+^ myeloid cells in the spleen into the peripheral circulation.

### CSD Promoted Tumor Proliferation and Invasion

**Figure 3:**
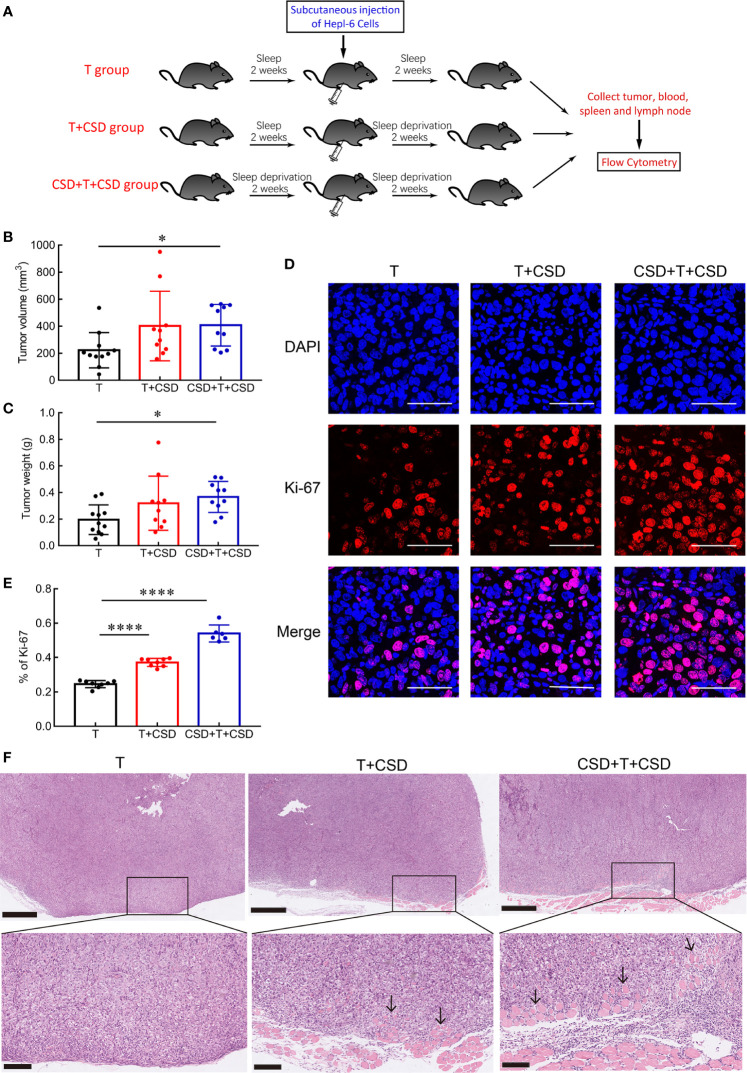
CSD promotes tumor growth, proliferation, and invasion. **(A)** Schematic illustration of the experimental groups. **(B, C)** CSD for 2 weeks before and after subcutaneous injection of Hep1-6 cells (CSD + T + CSD) significantly increased tumor size **(B)** and weight **(C)** compared to the normal sleep group (T). Mice in the T + CSD group were subjected to CSD for 2 weeks only after tumor implantation (n = 10–11 per group). **(D)** Representative immunofluorescence images of 4′,6-diamidino-2-phenylindole (DAPI)-stained nuclei (blue) and Ki-67 immunolabeling (red) in tumor sections from different groups and merged images at 630× magnification. Scale bars = 50 μm. **(E)** Quantification of the percentage of Ki-67-positive cells in tumor tissues from mice in the T, T + CSD, and CSD + T + CSD groups (n = 6–8 per group). **(F)** HE staining of subcutaneous tumor tissues. Tumor cells were observed to penetrate and infiltrate into adjacent muscle tissue in the T + CSD and CSD + T + CSD groups (arrows). Upper panels: 50×, scale bar = 500 μm; lower panels: 200×, scale bar = 100 μm. Data are presented as mean ± SEM. *p < 0.05, ****p < 0.0001.

The immune system is known to be involved in tumor initiation and development (8). Given that many patients with HC experience long-term sleep disturbance, we examined the influence of CSD on the development of HC using a mouse xenograft model in which HC cells (Hep1-6 cells) were subcutaneously injected into mice (**Figure 3A**). Tumor volume (**Figure 3B**) and weight (**Figure 3C**) were significantly increased in the CSD + T + CSD group compared to the T group (both *p* < 0.05) and were also higher in the T + CSD group compared to the T group, although the difference did not reach statistical significance. Representative images of subcutaneous tumors in the three groups are shown in **Supplementary Figure 1D**. These results indicate that CSD promotes the development of HC, and that the effect is dependent on the severity of SD.

To determine whether CSD accelerates tumor growth by enhancing the proliferation of tumor cells, we examined the expression of the proliferation marker Ki-67 (23) in tumor tissues by immunofluorescence analysis. Compared to the T group, the percentage of Ki-67-positive cells was significantly higher in the T + CSD and CSD + T + CSD groups (both *p* < 0.0001; **Figure 3D**), with a higher percentage in the latter than in the former (*p* < 0.0001; **Figure 3E**).

Tumor cell invasion and metastasis are the main causes of death from cancer (7). We performed a histological analysis to assess the effects of CSD on the invasive potential of subcutaneous HC cell tumors in mice by HE staining. Tumor cells infiltrated into adjacent muscle tissue in the T + CSD and CSD + T + CSD groups; however, the degree and depth of tumor cell invasion were greater in the latter. No obvious infiltration was observed in the T group (**Figure 3F**). These results indicate that CSD accelerates tumor growth and promotes the proliferation and invasion of HC, particularly when the CSD extends over a long period (before and after tumorigenesis).

### CSD Reduces Antitumor Immunity and Enhances Immunosuppression in the Tumor Microenvironment

**Figure 4:**
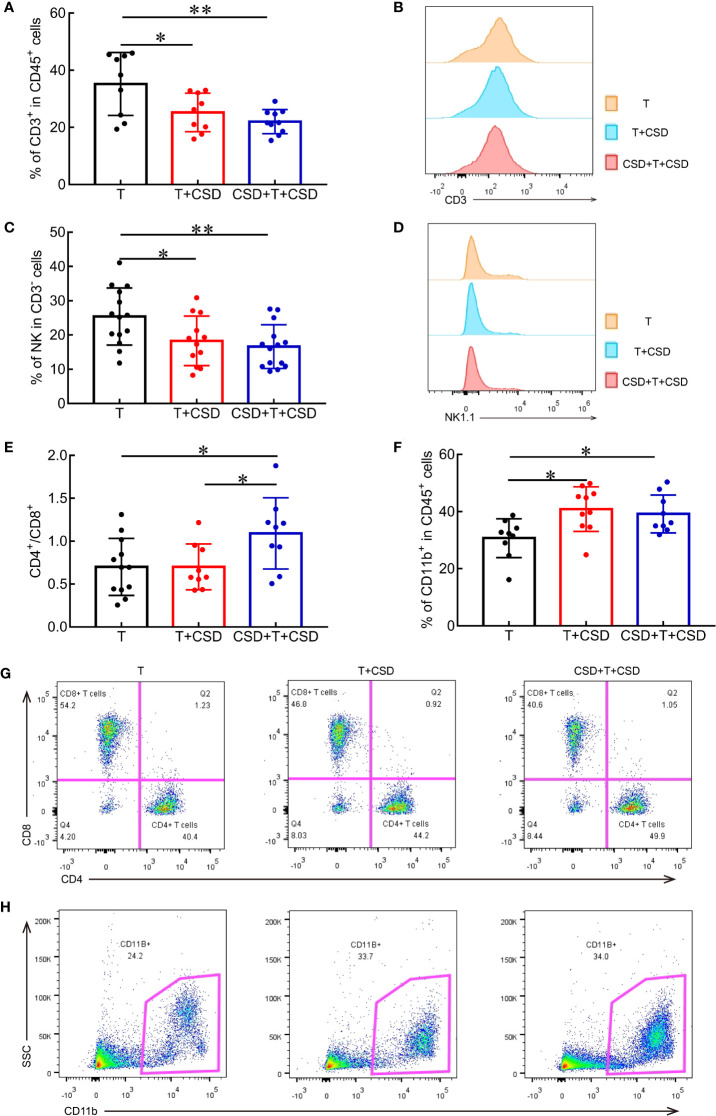
CSD reduces the infiltration of antitumor immune cells and increases that of immunosuppressive cells into the tumor microenvironment. **(A)** Quantification of the proportions of intratumor CD3^+^ T cells among CD45^+^ cells in the T, T + CSD, and CSD + T + CSD groups. **(B)** Representative histogram of CD3^+^ T cells in tumor tissues of each group. **(C)** Quantification of mean fluorescence intensity of NK cells infiltrating into tumor tissues. **(D)** Representative histogram of NK cells in the tumor microenvironment. **(E, F)** Quantitative analysis of flow cytometry results showing the CD4/CD8 ratio and percentages of CD11b^+^ cells within tumor tissues in each group. **(G, H)** Representative scatterplots of CD4^+^ T cells and CD8^+^ T cells **(G)** and CD11b^+^ cells **(H)** infiltrating into tumors in each group. Data represent mean ± SEM. *p < 0.05, **p < 0.01.

The tumor microenvironment supports the growth and metastasis of tumors (24). Various immune cell types infiltrate the tumor microenvironment including T lymphocytes (CD3^+^ T cells, CD4^+^ Th cells, and CD8^+^ cytotoxic T cells), NK cells, MDSCs, macrophages, and dendritic cells that are involved in tumor progression and related to cancer prognosis (25, 26). To investigate the role of immune cells in tumor progression induced by CSD, we quantified the proportions of immune cells in tumor tissues by multicolor flow cytometry analysis. CSD significantly decreased the percentages of antitumor CD45^+^CD3^+^ T lymphocytes (T *vs*. CSD + T, *p* = 0.0226; T *vs*. T + CSD + T, *p* = 0.0031; **Figures 4A, B**) and CD3^−^NK1.1^+^ NK cells (T *vs*. CSD + T, *p* = 0.0373; T *vs*. T + CSD + T, *p* = 0.0093; **Figures 4C, D**), indicating a reduced antitumor immune function (10–12). The CD4/CD8 ratio (T *vs*. CSD + T and T *vs*. CSD + T + CSD, both *p* < 0.05; **Figure 4E**) and proportion of immunosuppressive CD11b^+^ myeloid cells infiltrating into the tumor microenvironment were markedly increased in CSD mice (T *vs*. CSD + T + CSD and T + CSD *vs*. CSD + T + CSD, both *p* < 0.05; **Figure 4F**). However, no differences were observed in the percentages of intratumor Th cells (CD3^+^CD4^+^) among CD3^+^ cells (**Supplementary Figure 1F**) and cytotoxic T lymphocytes (CD3^+^CD8^+^) among CD3^+^ cells (**Figure 4G**, **Supplementary Figure 1G**) between the CSD and T groups.

MDSCs (specifically, PMN-MDSCs) were the main cells that were increased in the CD11b^+^ population in the tumor microenvironment of CSD mice, although the difference was non-significant (**Figure 4H**, **Supplementary Figures 1I, J**). However, CSD did not alter the proportions of macrophages and MN-MDSCs in tumor tissues (**Supplementary Figures 1H, K**). Thus, CSD decreased the infiltration of CD3^+^ T cells and NK cells and increased that of CD11b^+^ myeloid cells into tumor tissues, thereby suppressing antitumor immunity and enhancing immunosuppression in the tumor microenvironment, leading to increased tumor malignancy and progression.

### CSD Dysregulates Immune Cells in Peripheral Blood and Spleen of Tumor-Bearing Mice

**Figure 5:**
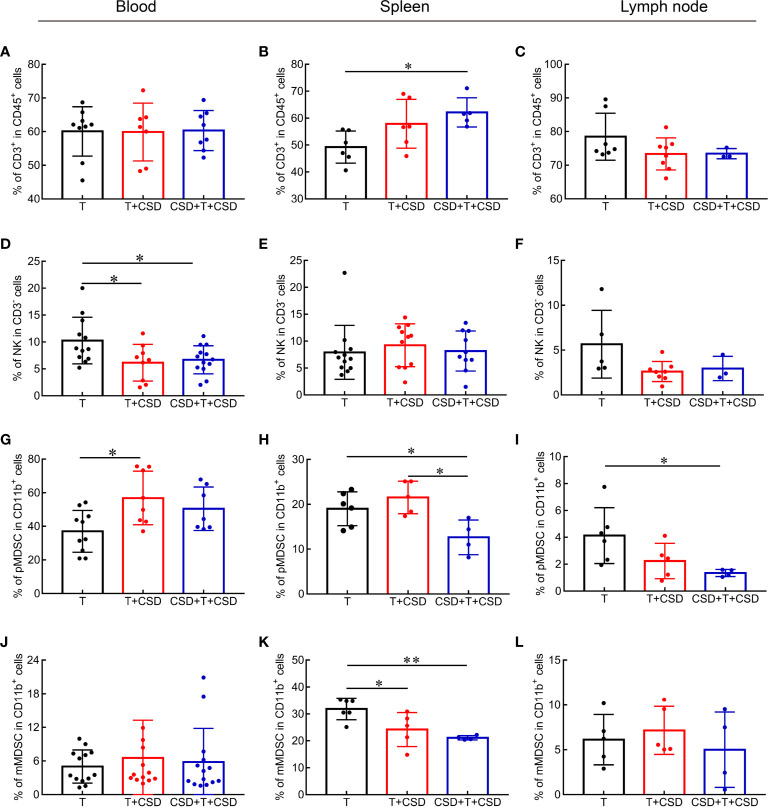
CSD redistributes immune cells in the circulation and peripheral immune organs (spleen and lymph node) in tumor-bearing mice. **(A–L)** CSD-induced changes in the percentages of CD3^+^ T cells **(A–C)**, NK cells **(D–F)**, PMN-MDSCs **(G–I)**, and MN-MDSCs **(J–L)** in blood **(A, D, G, J)**, spleen **(B, E, H, K)**, and lymph node **(C, F, I, L)** in tumor-bearing mice after CSD. Data are presented as mean ± SEM. *p < 0.05, **p < 0.01.

Local immune responses modulate systemic inflammation *via* signals secreted into the circulation that reach distant organs such as the spleen (27). We examined whether CSD induced changes in immune cells in the tumor microenvironment by promoting their systemic redistribution by measuring the percentages of immune cells in the blood, spleen, and lymph nodes of tumor-bearing mice. The proportion of CD3^+^ T cells in the spleen was significantly increased in the CSD + T + CSD group (*p* < 0.05), implying that the CSD-induced reduction in CD3^+^ T cells in the tumor microenvironment was due to a decrease in their mobilization from spleen to tumor (**Figures 5A–C**).

The percentage of NK cells in the blood was decreased (*p* < 0.05; **Figure 5D**), whereas these cells showed an increasing trend in the spleen and a decreased trend in the lymph node (**Figures 5E, F**), indicating that CSD caused their depletion from the tumor microenvironment by inducing their redistribution from the spleen through peripheral blood. No differences were detected in the abundance of CD4^+^ and CD8^+^ T cells or in the CD4/CD8 ratio in the blood and spleen between the T, T + CSD, and CSD + T +C SD groups (**Supplementary Figures 2A, B, D, E, G, H**), although the percentage of CD4^+^ T cells and CD4/CD8 ratio presented were significantly higher in the lymph node of the T + CSD group compared to the T group (**Supplementary Figures 2C, I**).

The percentages of CD11b^+^ cells showed an increasing trend in the blood and a decreasing trend in the spleen of mice in the CSD + T + CSD group (**Supplementary Figures 2S, T**). No obvious change was observed in the percentage of CD11b^+^ cells in the lymph node of tumor-bearing mice (**Supplementary Figure 2U**). The percentage of MDSCs in the blood was significantly increased (*p* < 0.05; **Supplementary Figure 2P**), while no changes were observed in macrophage populations in the blood and spleen upon CSD (**Supplementary Figures 2M, N**), indicating that MDSCs are the major subset of CD11b^+^ cells in the peripheral blood of tumor-bearing mice that were increased under this condition. PMN-MDSCs percentage was markedly elevated in peripheral blood (P<0.05; **Figure 5G**) while reduced in the spleen (T vs T+CSD and T vs CSD+T+CSD, both P<0.05; **Figure 5H**) and lymph node (**Figure 5I**). No changes of MN-MDSCs were observed in the blood or lymph node (**Figures 5J, L**) although the reduction of these cells was observed in the spleen (**Figure 5K**). Thus, PMN-MDSCs were the main type of MDSCs that were increased in the blood and decreased in the spleen by CSD. M-MDSCs were also decreased in the spleen of tumor-bearing mice (T *vs*. T + CSD and T *vs*. CSD + T + CSD, *p* < 0.05; **Figure 5K**). These results demonstrate that CSD promotes the infiltration of CD11b^+^ myeloid cell subsets (mainly PMN-MDSCs) into the tumor microenvironment in HC by inducing their redistribution from the spleen through the peripheral circulation to tumor tissues.

## Discussion

This study investigated the effects of ASD and CSD on tumor development and immune responses in HC. Both types of SD resulted in the perturbation of immune profiles in mice, causing similar changes in the proportions of immune cells in blood, spleen, and lymph node. Additionally, CSD promoted HC growth, proliferation, and invasion. Our results provide direct experimental evidence and suggest a mechanistic basis for the adverse effects of sleep disturbance on HC prognosis and underscore the interaction between the brain and immune system.

Sleep plays a critical role in regulating and maintaining immune homeostasis (28). SD has been shown to impair immunity (3, 29, 30). We found here that ASD significantly increased the percentages of CD3^+^ T lymphocytes in the blood and spleen, CD4^+^ Th cells in the blood and lymph node, CD4/CD8 ratio in the blood, and NK cells in the spleen and lymph node and decreased the proportion of cytotoxic CD8^+^ T cells in the blood. Meanwhile, CSD increased the percentages of CD3^+^ T cells in the spleen and lymph node, CD4^+^ Th cells in the blood, CD4/CD8 ratio in the blood and spleen, NK and NKT cells in the lymph node, and CD11b^+^ cells in the blood and decreased the proportion of CD8^+^ T cells and NKT cells in the spleen, suggesting that CSD promoted the recruitment of CD11b^+^ subsets (mainly MDSCs and PMN-MDSCs) from the spleen into the peripheral blood. In conclusion, ASD and CSD both induced increased CD3^+^ T cells, CD4^+^ Th cells, and NK cells and decreased CD8^+^ cytotoxic T cells in mice, which indicated that ASD and CSD could disturb immune surveillance in mice. In addition, NKT cells significantly changed in CSD mice but not in ASD mice. Notably, SD-induced changes in immune profiles were not limited to the blood but also involved peripheral organs, which could explain the increase in susceptibility to disease in individuals who experience long-term sleep disturbance including cancer patients.

Previous studies have reported a mechanistic link between SD and immune impairment that support our findings. Persistent wakefulness increased NK cell counts and activity in the circulation of healthy adults (31), while two nights of total SD increased CD4^+^ T cell numbers in healthy male volunteers (32). Paradoxical SD for 72 h decreased CD8^+^ T cell counts in mouse spleen (33). However, other reported changes in immune cell profiles induced by SD are inconsistent with our results. For example, 10 h of partial SD increased CD8^+^ T cell numbers, while no change was observed in CD3^+^ T cells (34). A reduced amount of sleep in healthy volunteers was associated with decreased NK cell activity (35), and NK and NKT cell numbers and cytotoxic activity were reduced after 72 h of paradoxical SD due to an increase in plasma glucocorticoids and downregulation of β_2_-adrenergic receptor in these cells (36). The conflicting data may be attributable to the different SD protocols that were used, sampling time, or genetic background of the study population. In our study, mice were subjected to total sleep deprivation (including both rapid and non-rapid eye movement sleep), whereas in some earlier work, only the former was targeted. In addition, SD could activate the hypothalamic–pituitary–adrenal axis to promote the release of glucocorticoids and promote the secretion of melatonin (37).Melatonin could shorten sleep latency and prolong sleep duration to promote sleep and regulate immune response. Considering that melatonin could simultaneously treat sleep deprivation and its inflammation, further studies about melatonin as a potential drug for the treatment of sleep deprivation were needed (38–40).

We found that CSD promoted the growth, proliferation, and invasion of HC by decreasing the redistribution of CD3^+^ T cells and NK cells and increasing the mobilization of CD11b^+^ subsets from the spleen to tumor tissues through the peripheral circulation. A previous study showed that SD reduced the number of CD8^+^ T cells in the spleen and the survival of mice with ascites tumors (41). Chronic sleep restriction was found to decrease the numbers of cytotoxic cells (CD8^+^ T cells and NK cells) in the tumor microenvironment of mice, thus impairing the antitumor immune response and accelerating the rate of lung metastasis (42). Intermittent hypoxia—a typical feature of obstructive sleep apnea—promoted the growth and invasion of mouse lung tumor cells induced by the transformation of tumor-associated macrophages to a protumorigenic M2 phenotype (43). However, these studies did not examine the effects of CSD on the distribution of immune cells in the tumor microenvironment and peripheral immune organs through the circulation in a tumor-bearing model. Additionally, the development of HC is associated with chronic inflammation and infection with HBV or hepatitis C virus or alcohol consumption (44, 45). Our mouse mode of SD simulated the experience of patients with sleep disturbances after and both before and after a diagnosis of liver cancer.

There were several limitations to our study. First, we used a heterotopic tumor model to investigate the effects of CSD and systemic immune dysregulation on HC progression. Experiments in orthotopic transplantation models of liver tumors such as chemically induced HC and spontaneous liver cancer are needed to validate our results. Second, we did not clarify the mechanisms by which CSD regulates immune cells in the tumor environment, blood, and peripheral immune organs. Finally, it remains unclear which signals (inflammatory cytokines or other molecules) secreted in the tumor microenvironment induce the systemic redistribution of immune cells.

In conclusion, we found that CSD reduced antitumor immunity and enhanced immunosuppression to promote the growth, proliferation, and invasion of HC through the redistribution of CD3^+^ T cells, NK cells, and CD11b^+^ subsets within the tumor environment and to peripheral immune sites (spleen and lymph node) through the circulation. Our findings highlight the importance of alleviating sleep disturbance in patients with liver cancer in order to reduce the risk of disease progression.

## Data Availability Statement

The original contributions presented in the study are included in the article/**Supplementary Material**. Further inquiries can be directed to the corresponding author.

## Ethics Statement

The animal study was reviewed and approved by Ethics Committee of School of Basic Medical Sciences, Fudan University.

## Author Contributions

CL: supervised the study and acquired funding. JH: designed the study, performed experiments and statistical analyses, and wrote the manuscript. PS, KH, ZC, ZZ, and YZ: assisted with animal experiments and data collection. JX and PW: assisted with *in vitro* experiments. JQ: analyzed the data. WQ and ZH: designed the experiments and revised the manuscript. All authors contributed to the article and approved the submitted version.

## Funding

This study was supported by the National Natural Science Foundation of China (grant no. 31971111 and 31471147).

## Conflict of Interest

The authors declare that the research was conducted in the absence of any commercial or financial relationships that could be construed as a potential conflict of interest.

## Publisher’s Note

All claims expressed in this article are solely those of the authors and do not necessarily represent those of their affiliated organizations, or those of the publisher, the editors and the reviewers. Any product that may be evaluated in this article, or claim that may be made by its manufacturer, is not guaranteed or endorsed by the publisher.
