# Supplementary material for: Sleep Deprivation Disturbs Immune Surveillance and Promotes the Progression of Hepatocellular Carcinoma
Source: Front Immunol. 2021 Sep 3;12:727959. doi: 10.3389/fimmu.2021.727959 (PMC8446513; doi:10.3389/fimmu.2021.727959)
Supplement: Supplementary file 1 [file DataSheet_1.docx]

Supplementary Material

Sleep deprivation disturbs immune surveillance and promotes the progression of hepatocellular carcinoma

**Jing Huang ^1^, Peiwen Song** **^3^, Kaibin Hang ^4^, Zeka Chen ^2^, Zidan Zhu ^1^, Yuye Zhang ^1^, Jietian Xu ^1^, Jie Qin ^1^, Binghua Wang ^1^, Meihong Qiu ^3^, Weimin Qu ^2^, Zhili Huang ^2^, Chunmin Liang** **^1^***

^1^ Laboratory of Tumor Immunology, Department of Anatomy, Histology, and Embryology, School of Basic Medical Sciences, Shanghai Medical College, Fudan University, Shanghai 200032, China

^2^ Department of Pharmacology, School of Basic Medical Sciences; State Key Laboratory of Medical Neurobiology and MOE Frontiers Center for Brain Science, and Institutes of Brain Science, Fudan University, Shanghai 200032, China

^3^ Department of Department of Neurobiology, School of Basic Medical Sciences, Fudan University, Shanghai 200052, China.

^4^ Department of Radiology, Naval Medical Center of People's Liberation Army, Shanghai 200052, China.

*** Correspondence:**Prof. Chunmin Liang
[cmliang@fudan.edu.cn](mailto:cmliang@fudan.edu.cn)

# Supplementary Figures and Tables

## Supplementary Figures


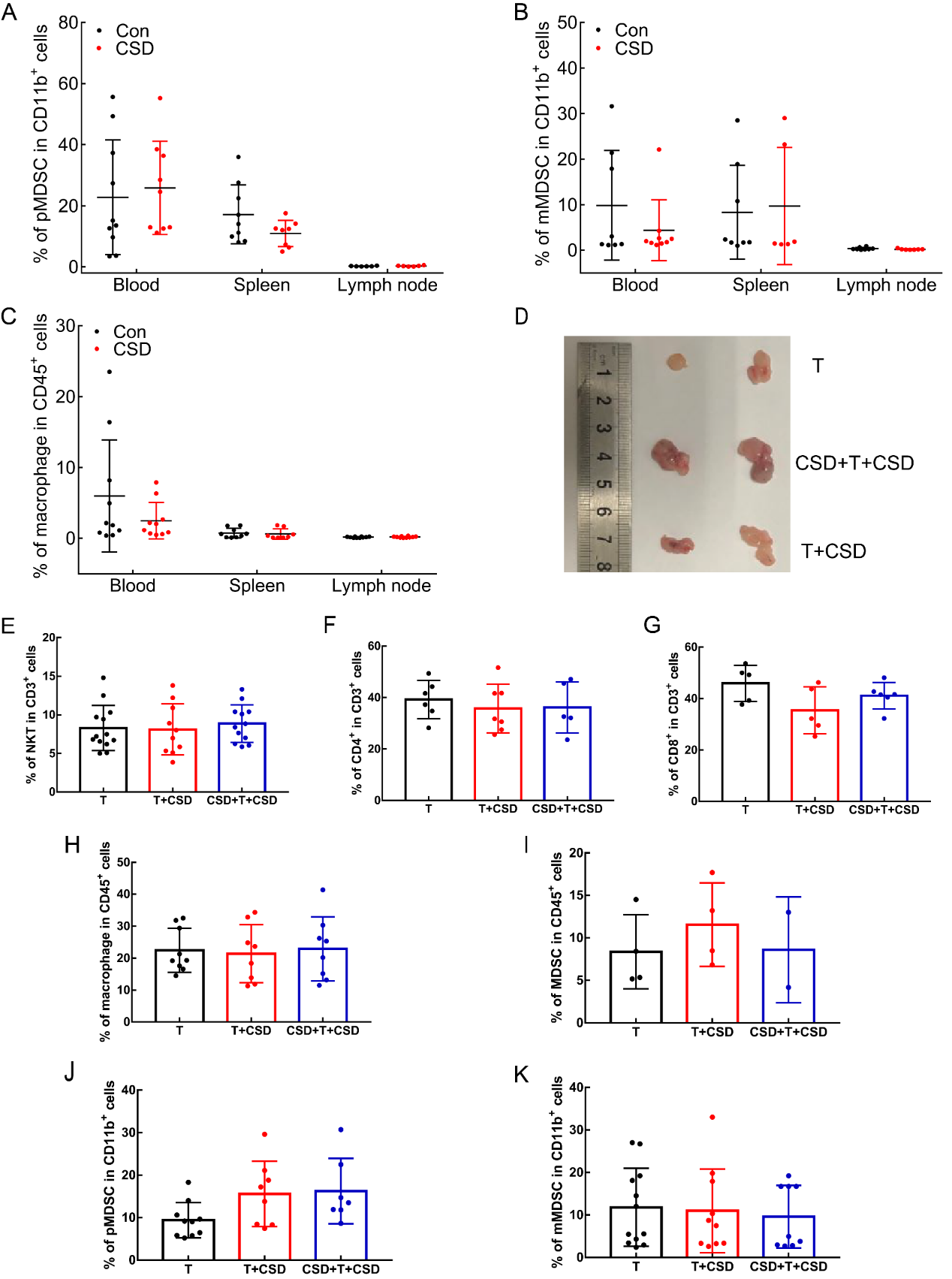
**Supplementary Figure 1.** **(A-C)** The effects of chronic sleep deprivation on the percentages of **(A)** PMN-MDSCs, **(B)** M-MDSCs and **(C)** macrophages in peripheral blood, spleen and lymph node of wild-type mice. **(D)** Representative images of subcutaneous tumor in T group, T+CSD group and CSD+T+CSD group. (E-K) Statistical graphs of the percentages of NKT cells **(E)**, CD4^+^ T cells **(F)**, CD8^+^ T cells **(G)**, macrophages **(H)**, MDSCs **(I)**, PMN-MDSCs **(J)** and M-MDSCs **(K)** in tumor tissues of T group, T+CSD group, CSD+T+CSD group.


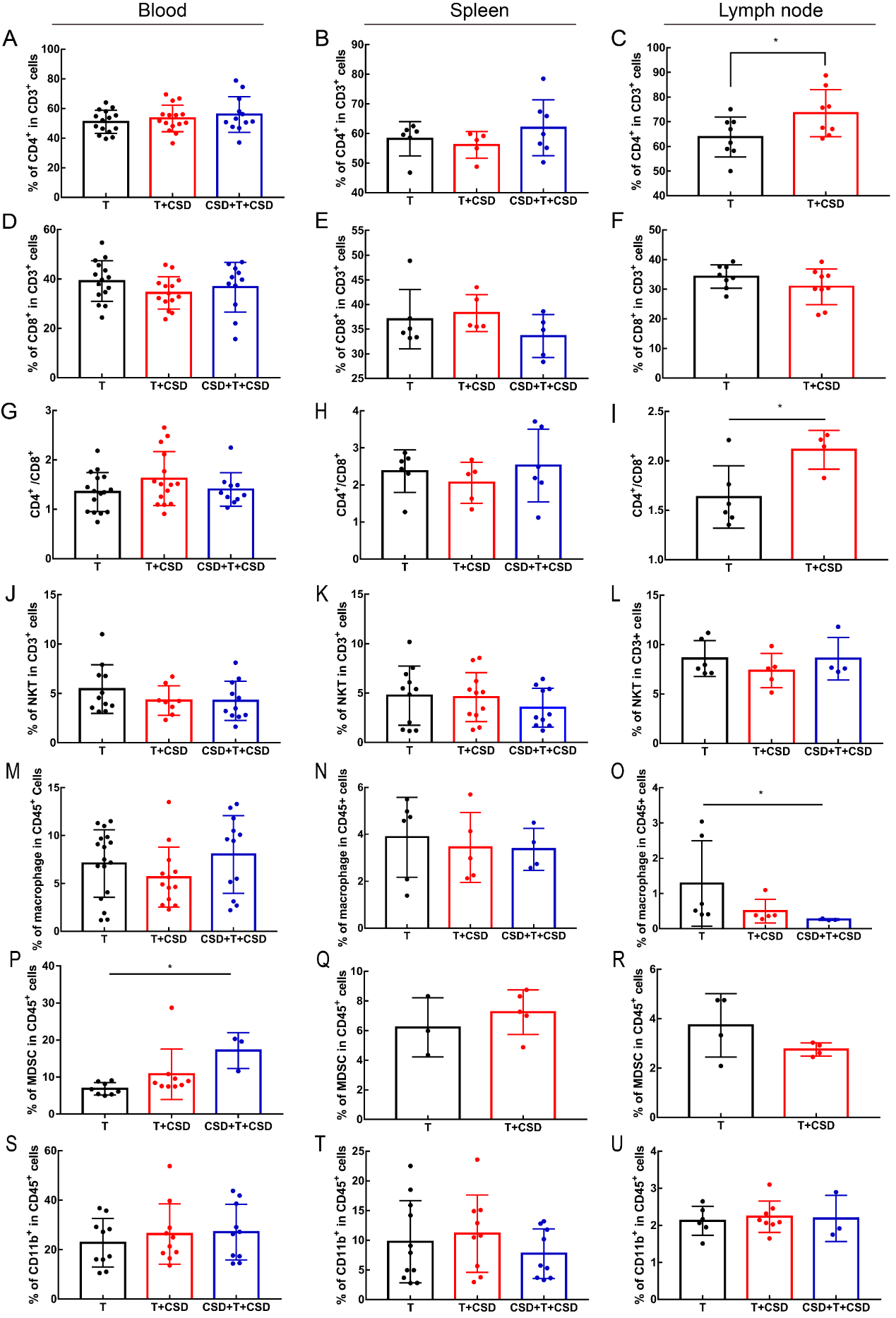
**Supplementary Figure 2. (A-C)** Statistical graphs of CD4^+^ T cells in blood **(A)**, spleen **(B)** and lymph node **(C)** of tumor-bearing mice from T group, T+CSD group and CSD+T+CSD group. **(D-F)** Statistical results of CD8^+^ T cells in blood **(D)**, spleen **(E)** and lymph node **(F)** of each group. **(G-I)** Comparisons of CD4/CD8 ratio between different groups in blood **(G)**, spleen **(H)** and lymph node **(I)**. **(J-L)** Statistical analysis of the percentages of NKT cells within CD3^+^ T cells in blood **(J)**, spleen **(K)** and lymph node **(L)** from tumor-bearing mice treated with chronic sleep deprivation. **(M-O)** Statistical results of the percentages of macrophages in blood **(M)**, spleen **(N)** and lymph node **(O)** from each group of mice. **(P-R)** Comparisons in the percentages of MDSCs in blood **(P)** between T, T+CSD and CSD+T+CSD group, spleen **(Q)** and lymph node **(R)** between T and T+CSD group. **(S-U)** Statistical graphs of CD11b^+^ cells in peripheral blood **(S)**, spleen **(T)** and lymph node **(U)** from different groups by flow cytometric analysis. *P<0.05.
